# Common Mental Disorders and Vitamin D Deficiency/Insufficiency: A Cross-Sectional Study Among Female Workers in Southern Brazil

**DOI:** 10.3390/nu18010025

**Published:** 2025-12-20

**Authors:** Ingrid Stähler Kohl, Anderson Garcez, Janaína Cristina da Silva, Harrison Canabarro de Arruda, Vera Maria Vieira Paniz, Maria Teresa Anselmo Olinto

**Affiliations:** 1 Post-Graduate Program in Medical Sciences: Endocrinology, Faculty of Medicine, Federal University of Rio Grande do Sul State, UFRGS, Porto Alegre 90610-264, RS, Brazil; ingridkohl.nutri@gmail.com (I.S.K.); adsgarcez@gmail.com (A.G.); 2 Post-Graduate Program in Food, Nutrition and Health, Faculty of Medicine, Federal University of Rio Grande do Sul, UFRGS, Porto Alegre 90610-264, RS, Brazil; harrisoncanabarro@gmail.com; 3 Post-Graduate Program in Collective Health, University of Vale do Rio dos Sinos, Unisinos, São Leopoldo 93022-000, RS, Brazil; cds.janaina@hotmail.com (J.C.d.S.); vpaniz@unisinos.br (V.M.V.P.)

**Keywords:** vitamin D, common mental disorder, shift work, workers’ health

## Abstract

**Background/Objectives**: The literature indicates that decreased vitamin D levels are frequently observed in individuals with severe psychiatric disorders. However, the scarcity of studies investigating this association in non-psychiatric populations, such as working women, limits the generalizability of these findings. Therefore, this study aimed to investigate the association between common mental disorders (CMDs) and vitamin D deficiency/insufficiency among female workers in southern Brazil. **Methods**: A cross-sectional study was conducted with a sample of 304 female workers from an industrial group in southern Brazil. Vitamin D deficiency/insufficiency was defined as a serum 25(OH)D concentration of <30 ng/mL. CMDs were assessed using the Self-Reporting Questionnaire (SRQ-20), with a cutoff score of ≥8. The association between CMDs and vitamin D deficiency/insufficiency was estimated using prevalence ratios (PRs) obtained through Poisson regression models adjusted for potential confounders. All analyses were stratified by age group (≤40 years and >40 years). **Results**: The ≤40-year group included 212 women (69.7%; mean age: 30.1 ± 6.3 years), and the >40-year group included 92 women (30.3%; mean age: 47.5 ± 5.6 years). The prevalence of vitamin D deficiency/insufficiency was 75.0% (95% confidence interval [CI]: 69.1–80.9) in women aged ≤40 years and 77.2% (95% CI: 68.4–85.9) in those aged >40 years. After adjustment for potential confounding variables, among women older than 40 years, those with CMDs had a 25% higher probability of presenting vitamin D deficiency/insufficiency compared to those without CMDs (PR = 1.25; 95% CI: 1.00–1.56; *p* = 0.044). Among women aged ≤40 years, no significant association was observed between CMDs and vitamin D deficiency/insufficiency (PR = 1.10; 95% CI: 0.94–1.30; *p* = 0.226). **Conclusions**: The findings of this study indicate a significant association between common mental disorders and vitamin D deficiency/insufficiency among female workers, particularly in those aged 40 years or older.

## 1. Introduction

The term common mental disorders (CMDs) encompasses symptoms of depression, anxiety, and somatization that—although causing significant distress—do not meet the formal diagnostic criteria outlined in manuals such as the DSM-5 or ICD-10 [1]. The World Health Organization defines CMDs as depressive and anxiety disorders, distinguishing them from normal emotional responses, such as sadness or stress, and highlighting their impact on individuals’ quality of life and well-being [2]. Globally, the prevalence of CMDs is estimated at 17.6% [3], whereas, in Brazil, the rates are higher, ranging from 20% to 56%, depending on the population studied. Higher frequencies have been reported among women and workers [4], with industrial workers, especially those who work shifts, constituting a particularly vulnerable group due to their occupational exposure profile and deregulated routines, which can elevate the risk for CMDs compared to the general population [4,5,6,7,8,9,10,11,12]. These findings are consistent with previous studies showing a greater prevalence among these population groups and an increase among older individuals [5,6,7,8,9,10,11,12].

Vitamin D status has been shown to influence a wide range of health outcomes [13,14]. Vitamin D deficiency is classically defined as a serum 25(OH)D concentration below 20 ng/mL (50 nmol/L), and insufficiency is defined as concentrations between 20 and 30 ng/mL (50–75 nmol/L) [15]. However, to achieve extra-skeletal benefits, the Endocrine Society recommends maintaining levels between 30 and 60 ng/mL, although optimal concentrations for specific health outcomes in healthy adults have not yet been established [16,17]. Globally, vitamin D deficiency and insufficiency are estimated to affect approximately 48% and 77% of the population, respectively [18], and they are more prevalent in women than in men (about 1.3 times higher), in older adults (about 2.5 times higher), and among other at-risk populations. Industrial workers, especially shift workers, are at high risk of inadequate vitamin D levels—approximately 2 times higher—primarily due to limited sun exposure resulting from working indoors and night shifts [18,19,20,21,22,23,24,25]. Therefore, this population represents a relevant group for specific investigation regarding this outcome.

Emerging evidence suggests a bidirectional relationship between vitamin D and mental health [26,27]. Several studies have associated lower serum 25(OH)D concentrations with depressive symptoms [28,29,30,31], although others have not confirmed this association [30,32,33]. Conversely, studies indicate that individuals with psychiatric disorders—including depression, schizophrenia, and phobias—often present with reduced vitamin D levels [34,35,36]. Nonetheless, the underlying mechanisms and health conditions involved in this relationship remain unclear. Furthermore, most of these studies were conducted in psychiatric or institutionalized populations, which may limit the generalizability of their findings.

When this relationship is analyzed in non-clinical and “healthy” populations, such as active workers, the available data are limited. Investigation among industrial workers is especially pertinent given their dual risk: greater vulnerability to both vitamin D deficiency and the development of CMDs. The existing evidence in these populations is not only scarce but also inconsistent. For example, a South Korean study reported a higher likelihood of depressive symptoms among individuals with severe vitamin D deficiency (<10 ng/mL) [37]. Conversely, a study of Taiwanese nurses found no significant association between vitamin D levels and mental health parameters, including depressive symptoms, sleep problems, and fatigue [38]. However, it is important to note that both studies have methodological limitations, including their cross-sectional design, which prevents establishing causality; their focus on specific population profiles, which render it difficult to generalize the results; and their use of self-reported measures for mental health outcomes, which may introduce reporting bias.

Considering this evidence gap and the methodological limitations of prior research, along with the greater vulnerability of industrial workers—especially women in regions with lower sunlight exposure—to vitamin D deficiency and CMDs, the present study aims to (1) investigate whether an association exists between 25-hydroxyvitamin D deficiency/insufficiency and common mental disorders (CMDs) among active industrial female workers in southern Brazil, and (2) evaluate whether age acts as an effect modifier in this relationship. We hypothesize that the prevalence of vitamin D deficiency/insufficiency is higher among female workers with CMDs and that this relationship is stronger among those in older age groups.

## 2. Materials and Methods

### 2.1. Study Design and Population

This was a cross-sectional study conducted with a sample of female workers from a large industrial group in the plastics and household utensils sector, located in the metropolitan area of Porto Alegre, Rio Grande do Sul State, Brazil. The present study is part of a broader research project entitled “Health Conditions of Women Shift Workers: Longitudinal Study of Occupational Health (ELO Saúde)”, which was submitted to and approved by the Research Ethics Committee of the University of Vale do Rio dos Sinos (CAAE No. 53762521.7.0000.5344; Opinion No. 5681627). All participants provided written informed consent in accordance with the ethical principles of the Declaration of Helsinki, including respect for autonomy, confidentiality, and anonymity.

### 2.2. Sample and Sampling

All female workers aged 18 years or older from three factories belonging to the same industrial group specializing in the manufacture of household plastic utensils were considered eligible for inclusion. Employees from both the production and administrative sectors were invited to participate in this study. Exclusion criteria included pregnancy at any stage, temporary leave from work, or employment duration of less than three months.

Of the 546 eligible female workers, 452 agreed to participate in the interview. All participants were invited to undergo blood collection, and laboratory data were obtained from 304 workers—232 from the production sector (fixed shifts; six-day work week; one day off) and 72 from the administrative sector (five-day work week; two days off).

### 2.3. Data Collection and Instruments

Data collection was carried out between August 2022 and March 2023. A standardized, pre-coded, and previously tested questionnaire was administered through face-to-face interviews conducted at the participants’ workplaces. All interviewers received specific training, and a pilot study was conducted to evaluate the instruments and train the research team. To ensure data quality, 10% of the interviews were repeated via telephone using a shortened version of the questionnaire that included items with stable responses over time.

Laboratory analyses were performed by a certified company, and blood samples were collected by trained professionals either at the workplace (in a designated private area) or at the participants’ homes. Participants were instructed to fast for a minimum of 8 h and a maximum of 12 h; abstain from alcohol consumption for at least 72 h; avoid caffeine intake; and refrain from engaging in vigorous physical activity within 24 h prior to sample collection. All collected data were coded and verified by research supervisors.

### 2.4. Outcome: Vitamin D Deficiency/Insufficiency

Serum concentrations of vitamin D [25(OH)D] were determined from serum samples with a minimum volume of 2.0 mL. The samples were centrifuged at 5000 rpm for 10 min at 18 °C and analyzed using the Microparticle Chemiluminescent Immunoassay (CMIA) method, with results expressed in ng/mL.

The outcome variable was defined according to the criterion established by the Endocrine Society, which classifies vitamin D deficiency/insufficiency as a serum level of <30 ng/mL [39]. The adoption of this cutoff point is justified because it allows for a more sensitive and earlier identification of suboptimal vitamin D status, aligning with a preventive approach aimed at optimizing the physiological functions of vitamin D. Although vitamin D deficiency (values < 20 ng/mL) is clearly associated with bone disorders such as rickets and osteomalacia, insufficiency (levels between 20 and 29.9 ng/mL) represents a subclinical risk condition. Even among apparently healthy individuals, this range has been associated with metabolic alterations such as increased parathyroid hormone (PTH) secretion, as well as potential risks for certain types of cancer, musculoskeletal problems (including chronic pain and muscle weakness), and immune dysfunctions [40,41,42].

### 2.5. Main Exposure: Common Mental Disorders (CMDs)

The presence of common mental disorders (CMDs) was assessed using the Self-Reporting Questionnaire (SRQ-20) [43]. This self-report instrument is widely employed for the early detection of signs and symptoms of non-psychotic mental disorders. It consists of 20 close-ended questions with dichotomous response options (yes/no), designed to screen for depression, anxiety, insomnia, fatigue, irritability, forgetfulness, difficulty concentrating, and physical complaints during the previous 30 days. The SRQ-20 has been validated for use in Brazilian Portuguese, with a cutoff score of ≥8 positive responses (SRQ-20 ≥ 8) indicating the presence of CMDs among adult women [43]. In addition, the instrument has demonstrated applicability in the assessment of mental health within occupational contexts [44]. Internal consistency analyses in the present study revealed satisfactory reliability (Kuder–Richardson coefficient [KR-20] = 0.859). In this cross-sectional study, women without CMDs (SRQ-20 < 8) were considered the reference group for all comparisons, serving as an internal control within each age stratum.

### 2.6. Explanatory Variables (Covariates)

Demographic, socioeconomic, behavioral, reproductive, health, and occupational data were collected to characterize the study population and to control for potential confounding variables in the multivariate analyses. Demographic and socioeconomic characteristics included the following: age (in complete years at the time of the interview) categorized into two age groups (≤40 years and >40 years); self-reported skin color categorized as White or Others (Black, Brown, Indigenous, or Yellow); marital status (without a partner—single, separated, divorced, or widowed; with a partner—married or in a union); educational attainment in completed years of study (≤8, 9–11, and ≥12 years); and per capita family income, calculated as the sum of the reported income of all household members during the previous month divided by the number of residents and categorized by the number of minimum wage earners (<1, ≥1–≤2, and >2 minimum wage earners; considering the 2022 national minimum wage of BRL 1212.00). Household headship was determined by whether the participant reported being financially responsible for the family (classified as “no” or “yes”). Behavioral characteristics included the following: leisure-time physical activity, based on self-reported engagement in physical activity, sports, or exercise during the previous week (excluding commuting) and categorized as “no” or “yes”; smoking status (non-smoker, former smoker, and current smoker); alcohol consumption (no—no intake or intake less than once per week; yes—at least once per week in the previous year); fruit and vegetable consumption (no—no intake; yes—intake of fruits and/or vegetables in the week preceding the interview); and usual sleep duration, based on reported daily hours of sleep during the week and categorized as “>6 h per day” or “≤6 h per day.” Reproductive characteristics included menstruation in the past 12 months (absence or presence of at least one menstrual period, categorized as “no” or “yes”) and the number of pregnancies (0, 1, or ≥2). Health characteristics included the presence of general obesity, assessed using body mass index (BMI) and calculated as the mean of two body weight measurements (kg) divided by the mean of two height measurements (m^2^). Obesity was defined as BMI ≥30 kg/m^2^ [45]. Occupational characteristics included work shift, determined from reported work start and end times and categorized as “day shift” (06:00 a.m. ≤ time < 10:00 p.m.) and “night shift” (10:00 p.m. ≤ time < 06:00 a.m.).

### 2.7. Statistical Analyses

Data entry was performed using EpiData software, version 3.1 (Centers for Disease Control and Prevention, Atlanta, GA, USA), following a double-entry procedure with subsequent comparison and consistency verification between datasets. Descriptive statistics were used to characterize the sample and describe the distribution of the main exposure (CMDs) and the outcome variable (vitamin D deficiency/insufficiency). Numerical variables were presented as means and standard deviations, whereas categorical variables were expressed as absolute (n) and relative (%) frequencies. Bivariate analyses were conducted to assess associations between variables. Student’s *t*-test was performed to compare the means of numerical variables, and Pearson’s chi-square test was used to assess the heterogeneity of proportions for dichotomous and nominal categorical variables. The *p*-value for linear trends was used for ordinal categorical variables. To examine the association between CMDs and vitamin D deficiency/insufficiency, prevalence ratios (PRs) and corresponding 95% confidence intervals (95% CIs) were estimated using Poisson regression with robust variance [46]. Five analytical models were constructed based on a hierarchical conceptual framework for variable inclusion in the adjusted analyses [47]: Model I: unadjusted (crude) analysis; Model II: adjusted for demographic and socioeconomic characteristics; Model III: adjusted for Model II plus behavioral characteristics; Model IV: adjusted for Model III plus reproductive and occupational characteristics; and Model V: adjusted for Model IV plus obesity. Variables with a significance level of ≤20% (*p* ≤ 0.20) in the bivariate analysis related to either the exposure or outcome were included in the multivariate models (Models II–V). All analyses were stratified by age group (≤40 years and >40 years). The Mantel–Haenszel test was applied to assess potential effect modification by age group in the relationship between vitamin D status and CMDs (*p* = 0.022).

All analyses were conducted using Stata software, version 14.0 (StataCorp LP, College Station, TX, USA). Statistical significance was set at a *p*-value of <0.05.

## 3. Results

A total of 304 female workers aged 18–64 years (mean 35.4 ± 10.1 years) were included in the final analysis. Table 1 shows the sample characteristics and the prevalence of common mental disorders (CMDs) and vitamin D deficiency/insufficiency, stratified by age group (≤40 and >40 years). The ≤40-year group comprised 212 women (69.7%; mean 30.1 ± 6.3 years), and the >40-year group comprised 92 women (30.3%; mean 47.5 ± 5.6 years). Most participants were White (68.4% and 72.8%), had 9–11 years of schooling (50.0% and 60.5%), reported family income from 1 to 2 minimum wage earners (41.5% and 42.4%), and reported no leisure-time physical activity (68.9% and 73.9%) or smoking (76.9% and 75.0%). Day-shift work predominated (89.2% and 81.5%). Compared with younger women, those aged >40 years were more often heads of household (45.7% vs. 30.2%), consumed less alcohol (20.6% vs. 35.8%), had lower educational attainment (≥12 years: 25.0% vs. 45.7%), and reported menstruation in the past year more frequently (48.9% vs. 20.3%).

The overall prevalence of CMDs was 46.4% (95% CI: 40.7–52.0), higher among women ≤40 years (50.0%; 95% CI: 43.2–56.8) than those >40 years (38.0%; 95% CI: 28.0–48.1; *p* = 0.055). CMD prevalence was greater among smokers in both age groups and among night-shift workers aged >40 years (58.8% vs. 33.3%; *p* = 0.051). Vitamin D deficiency/insufficiency was observed in 75.7% (95% CI: 70.8–80.5) of participants—75.0% (95% CI: 69.1–80.5) among those ≤40 years and 77.2% (95% CI: 66.4–85.9) among those >40 years (*p* = 0.685). In women aged >40 years, prevalence was higher among those sleeping ≤6 h per day (86.7% vs. 68.1%; *p* = 0.034) and marginally higher among night-shift workers (94.1% vs. 73.3%; *p* = 0.065). Among women aged ≤40 years, deficiency/insufficiency was lower in those with obesity (64.5% vs. 79.3%; *p* = 0.023) (Table 1).

As shown in Table 2, a significant association between CMDs and vitamin D deficiency/insufficiency was observed only in women aged >40 years. After full adjustment (Model V), those with CMDs had a 25% higher probability of presenting vitamin D deficiency/insufficiency than those without CMDs (PR = 1.25; 95% CI: 1.01–1.56; *p* = 0.044). No significant association was found among women ≤40 years (PR = 1.10; 95% CI: 0.94–1.30; *p* = 0.226) (Table 2).

## 4. Discussion

This study examined the association between common mental disorders (CMDs) and vitamin D deficiency/insufficiency among female workers from a large industrial group in southern Brazil. A higher probability of vitamin D deficiency/insufficiency was observed among women with CMDs, particularly in those aged over 40 years. Although menopausal status and sex hormone levels were not directly assessed, the age-stratified findings suggest that hormonal changes related to the climacteric period may partly explain the observed association among women over 40 years of age.

The higher likelihood of vitamin D deficiency/insufficiency observed among female workers with CMDs is a novel finding in the literature. To our knowledge, only one prior study has specifically examined this relationship among workers and found no significant association [38]. In contrast, studies involving psychiatric patients—both in inpatient and outpatient settings—frequently report a high prevalence of vitamin D deficiency. In these populations, deficiency is mainly associated with the presence of severe mental disorders such as schizophrenia, Alzheimer’s disease, dementia, and phobias; prolonged hospitalization (≥1 year); and the use of psychotropic medications, such as clozapine. This antipsychotic, commonly prescribed for schizophrenia, is known to cause various endocrine and hematological side effects, and it is metabolized by the hepatic cytochrome P450 system, potentially interfering with enzymes involved in vitamin D metabolism [48]. However, such studies typically compare psychiatric patients with the general population [36,49,50].

Our main hypothesis to explain the higher prevalence of vitamin D deficiency/insufficiency among workers with CMDs lies in the typical behavioral patterns associated with these disorders. Individuals with depressive or anxiety symptoms commonly exhibit social withdrawal, fatigue, reduced activity, sleep and appetite disturbances, and loss of interest in daily activities, among other manifestations [51]. This condition is often compounded by reduced health self-care and limited access to healthcare services [52,53]. Data from the 2014 Adult Psychiatric Morbidity Survey (APMS) showed that only 37% of individuals with CMDs were receiving any form of treatment at the time of assessment [54]. These factors contribute to greater isolation and restricted social routines, resulting in reduced sunlight exposure—the primary source of vitamin D—and poorer dietary habits, ultimately resulting in lower serum concentrations of this micronutrient.

The mechanism by which vitamin D influences mental health has been extensively studied and debated in recent years. Vitamin D receptors are abundantly expressed in several brain regions, including the prefrontal cortex, hippocampus, and hypothalamus, suggesting their involvement in regulating cognitive and affective processes [55]. Moreover, vitamin D participates in the regulation of neurotransmitters such as serotonin and melatonin, directly affecting mood and sleep patterns [56]. Consequently, inadequate vitamin D levels may impair neurochemical function and increase susceptibility to depressive and anxiety symptoms. These symptoms, in turn, promote social withdrawal and unhealthy behaviors that further reduce sunlight exposure and worsen dietary quality, creating a self-perpetuating cycle that deteriorates both nutritional status and mental health.

Our study found that the association between CMDs and vitamin D deficiency/insufficiency was evident only in women aged over 40 years. In this group, age may act as a risk factor that amplifies the previously described self-perpetuating cycle, further aggravated by additional elements such as estrogen decline and the onset of chronic medication use. Several drug classes commonly prescribed in this population may contribute to reduced vitamin D levels, either by inducing the enzyme CYP3A4—which accelerates vitamin D degradation—or by impairing gastrointestinal absorption. These include angiotensin-converting enzyme (ACE) inhibitors, statins, antibiotics, bile acid sequestrants, proton pump inhibitors (PPIs), corticosteroids, laxatives, thiazide diuretics (which may cause hypercalcemia but are also associated with vitamin D depletion), and lipase inhibitors [57,58]. In addition, the hormonal decline characteristic of the climacteric period exerts a significant influence. Estrogen plays a crucial role in the activation of vitamin D, particularly by regulating its binding protein (vitamin D binding protein, VDBP). Consequently, decreased estrogen levels may lower the free, biologically active fraction of serum vitamin D [59,60]. Therefore, the combined effects of aging, polypharmacy, and endocrine changes may create a high-risk scenario for vitamin D deficiency and related neuroendocrine disturbances in this population.

Our study found a vitamin D deficiency/insufficiency prevalence of 75.7% (95% CI: 70.8–80.5). This high rate aligns with previous research using the <30 ng/mL threshold and with studies conducted among indoor workers, who consistently exhibit higher prevalence rates than the general population [18,61,62]. Regarding age, no significant difference was observed between groups (75.0% vs. 77.2%, *p* = 0.685). Although a systematic review reported a lower prevalence of deficiency and insufficiency in individuals aged 45–64 years compared with those aged 19–44 years [18], this pattern was not evident in our sample. The authors attributed this finding to the more frequent use of vitamin D supplements among older adults, which may explain the discrepancy between their results and ours [18,63,64]. In general, advancing age is associated with reduced vitamin D synthesis and altered metabolism, largely due to a decline in cutaneous 7-dehydrocholesterol, which limits calcidiol production [20]. Chalcraft et al. quantified the photoconversion response of vitamin D metabolites after sun exposure in younger and older adults, showing a 13% decrease per decade and approximately half the production at age 70 compared with age 20 [65]. Considering these factors, a lower prevalence of vitamin D deficiency/insufficiency in the older age group was not expected. However, our study population is relatively young; the group aged >40 years, although including women up to 64 years, had a mean age of only 47.5 (±5.6) years, which may not be sufficiently advanced for age-related decline to fully manifest.

Conversely, the prevalence of CMDs in our study differed slightly between age groups, approaching statistical significance (50.0% vs. 38.0%, *p* = 0.055), suggesting a trend toward higher rates among younger women. The 2017 Global Burden of Disease (GBD) study and other reports have documented higher CMD prevalence among older adults in the general population, typically attributed to comorbidities, loss of autonomy, and social isolation [2,66,67,68]. The apparent contradiction may be partly explained by the “healthy worker effect.” This selection bias arises because individuals with disabling chronic conditions are less likely to remain in the labor force, either due to job dismissal, extended sick leave, or inability to enter employment. Consequently, this may mask the true prevalence of CMDs in older age groups, creating the illusion that older workers are more mentally resilient when, in reality, those with poorer health have already been excluded from occupational activities [69]. In contrast, among younger women, factors such as dual workloads, productivity pressure, childcare responsibilities, and financial instability may represent significant risk factors for CMDs [70,71]. This supports our observation that lower income was associated with CMDs only among women under 40 years of age, underscoring that the social determinants of CMDs may vary across life stages and socioeconomic contexts.

Among the strengths of our study, it is one of the first to examine the association between CMDs and vitamin D status in a non-psychiatric population, focusing on a specific sample of female industrial workers in southern Brazil. Standardized and certified procedures were used for vitamin D collection and analysis, along with a validated instrument for assessing the presence of CMDs [43,44]. Data were obtained through face-to-face interviews, which also included the assessment of several potential confounding factors and detailed occupational information. However, certain limitations should be acknowledged. Although our findings suggest an association between CMDs and vitamin D deficiency/insufficiency, the cross-sectional design precludes establishing causality. This study also did not assess the use of cholecalciferol supplements, duration of sun exposure, medical diagnoses of comorbidities, or medication use, preventing a more detailed analysis of these potential confounders. Furthermore, menopausal status and circulating sex hormone levels were not assessed, which limited our ability to formally examine the potential influence of hormonal changes on the age-specific associations observed. Finally, because the sample consisted of factory workers, the generalizability of our results to other populations may be limited due to the specific characteristics of this occupational group.

## 5. Conclusions

The findings of this study reveal a significant association between common mental disorders (CMDs) and vitamin D deficiency/insufficiency among female workers in a large industrial group in southern Brazil, particularly among those aged over 40 years. These results suggest that this occupational group is at increased risk and highlight the need for greater attention and health monitoring. Confirmations of this association in future longitudinal studies are essential to strengthen the scientific basis for developing targeted interventions and prevention strategies against hypovitaminosis D, including screening and supplementation programs. However, the implementation of vitamin D supplementation strategies should be accompanied by appropriate clinical monitoring to prevent potential vitamin D overload and associated adverse effects. Establishing safe dosage thresholds and follow-up protocols is crucial to ensure both the effectiveness and safety of preventive and therapeutic interventions.

## Figures and Tables

**Table 1:** General sample characteristics and prevalence of common mental disorders (CMDs) and vitamin D deficiency/insufficiency (<30 ng/mL) according to demographic, socioeconomic, behavioral, reproductive, health, and occupational characteristics—stratified by age group (≤40 years and >40 years)—among female workers in southern Brazil, 2022 (N = 304).

Features			CMDs (SRQ-20 ≥ 8)				Vitamin D (<30 ng/mL) ^a^			
	Total (304)		141 (46.4%)				230 (75.7%)			
	≤40 years	>40 years	≤40 years		>40 years		≤40 years		>40 years	
	n (%)	n (%)	n (%)	*p*-Value ^c^	n (%)	*p*-Value ^c^	n (%)	*p*-Value ^c^	n (%)	*p*-Value ^c^
Total	212 (69.7)	92 (30.3)	106 (50.0)		35 (38.0)		159 (75.0)		71 (77.2)	
Age, years (MD, SD) *	30.1 (6.3)	47.5 (5.6)	28.9 (6.4)		46.4 (4.5)	≤0.001 ^b^	29.7 (6.2)		47.7 (5.3)	≤0.001 ^b^
Skin color				0.883		0.805		0.348		0.341
White	145 (68.4)	67 (72.8)	73 (50.3)		26 (38.8)		106 (73.1)		50 (74.6)	
Others	67 (31.6)	25 (27.2)	33 (49.2)		9 (36.0)		53 (79.1)		21 (84.0)	
Marital status				0.679		0.337		0.690		0.663
No companion	117 (55.2)	40 (43.5)	60 (51.3)		13 (32.5)		89 (76.1)		30 (75.0)	
With partner	95 (44.8)	52 (56.5)	46 (48.4)		22 (42.3)		70 (73.7)		41 (78.8)	
Education (years) *				0.523		0.617		0.840		0.343
≤8	9 (4.2)	13 (14.1)	6 (66.7)		6 (46.1)		6 (66.7)		8 (61.5)	
9–11	106 (50.0)	56 (60.9)	54 (51.0)		22 (39.3)		80 (75.5)		45 (80.4)	
≥12	97 (45.7)	23 (25.0)	46 (47.4)		7 (30.4)		73 (75.3)		18 (78.3)	
Per capita family income (n = 303) ^d^				0.050		0.492		0.976		0.632
<1 SM	82 (38.7)	30 (32.6)	43 (52.4)		14 (46.7)		62 (75.6)		22 (73.3)	
1–2 MW	88 (41.5)	39 (42.4)	49 (55.7)		13 (33.3)		66 (75.0)		32 (82.0)	
>2 SM	42 (19.8)	23 (25.0)	14 (33.3)		8 (34.8)		31 (73.8)		17 (73.9)	
Head of household *				0.765		0.394		0.490		0.837
No	148 (69.8)	50 (54.3)	73 (49.3)		21 (42.0)		113 (76.3)		39 (78.0)	
Yes	64 (30.2)	42 (45.7)	33 (51.6)		14 (33.3)		46 (71.9)		32 (76.2)	
Physical activity (leisure)				0.373		0.126		0.123		0.768
No	146 (68.9)	68 (73.9)	76 (52.0)		29 (42.6)		114 (78.1)		53 (77.9)	
Yes	66 (31.1)	24 (26.1)	30 (45.4)		6 (25.0)		45 (68.2)		18 (75.0)	
Smoking				<0.001		0.027		0.871		0.845
Never smoked	163 (76.9)	69 (75.0)	70 (42.9)		21 (30.4)		121 (74.2)		53 (76.8)	
Former smoker	37 (17.4)	16 (17.4)	24 (64.9)		9 (56.2)		29 (78.4)		12 (75.0)	
Smoker	12 (5.7)	7 (7.6)	12 (100)		5 (71.4)		9 (75.0)		6 (85.7)	
Alcohol consumption *				0.567		0.682		0.186		0.307
No consumption	136 (64.2)	73 (79.4)	66 (48.5)		27 (37.0)		106 (77.9)		58 (79.4)	
≥1 time a week	76 (35.8)	19 (20.6)	40 (52.6)		8 (42.1)		53 (69.7)		13 (68.4)	
Fruit/vegetable consumption				0.528		0.961		0.203		0.820
No	54 (25.5)	16 (17.4)	25 (46.3)		6 (37.5)		37 (68.5)		12 (75.0)	
Yes	158 (74.5)	76 (82.6)	81 (51.3)		29 (38.2)		122 (77.2)		59 (77.6)	
Usual sleep hours (day week)				0.129		0.959		0.474		0.034
>6	115 (54.3)	47 (51.1)	52 (45.2)		18 (38.3)		84 (73.0)		32 (68.1)	
≤6	97 (45.7)	45 (48.9)	54 (55.7)		17 (37.8)		75 (77.3)		39 (86.7)	
Menstruating in the last 12 months *				0.393		0.959		0.375		0.104
Yes	43 (20.3)	45 (48.9)	24 (55.8)		17 (37.8)		30 (69.8)		38 (84.4)	
No	169 (79.7)	47 (51.1)	82 (48.5)		18 (38.3)		129 (76.3)		33 (70.2)	
Number of pregnancies				0.298		0.089		0.085		0.908
0	97 (45.7)	15 (16.3)	46 (47.4)		4 (26.7)		72 (74.2)		11 (73.3)	
1	58 (27.4)	30 (32.6)	34 (58.6)		8 (26.7)		49 (84.5)		23 (76.7)	
≥2	57 (26.9)	47 (51.1)	26 (45.6)		23 (48.9)		38 (66.7)		37 (78.7)	
Obesity (BMI > 30 kg/m^2^)				0.546		0.316		0.023		0.572
No	150 (70.7)	61 (66.3)	77 (51.3)		21 (34.4)		119 (79.3)		46 (75.4)	
Yes	62 (29.2)	31 (33.7)	29 (46.8)		14 (45.2)		40 (64.5)		25 (80.6)	
Work shift				0.508		0.051		0.161		0.065
Day shift	189 (89.2)	75 (81.5)	96 (50.8)		25 (33.3)		139 (73.5)		55 (73.3)	
Night shift	23 (10.8)	17 (18.5)	10 (43.5)		10 (58.8)		20 (87.0)		16 (94.1)	

MD, Mean; SD, standard deviation; SM, minimum wage; alcohol consumption, referring to the last year; consumption of fruits/vegetables, referring to the last week; ‘Others’ skin color: Black, Brown, Indigenous, or Yellow; BMI, body mass index. * *p*-value ≤ 0.05 for Pearson’s chi-square test for heterogeneity of proportions between age groups (≤40 years and >40 years). ^a^ Vitamin D deficiency/insufficiency (Endocrine Society) (Holick et al., 2011 [39]). ^b^ Student’s *t*-test for comparison of means. ^c^ Pearson’s Chi-square test for heterogeneity of proportions. ^d^ Total N differs due to loss of information (missing data).

**Table 2:** Unadjusted and adjusted prevalence ratios (PRs) and their respective 95% confidence intervals (95% CIs) for the association between common mental disorders (CMDs) and vitamin D deficiency/insufficiency (<30 ng/mL)—stratified for age (≤40 years and >40 years)—among female workers in southern Brazil, 2022 (N = 304).

CMDs	Vitamin D (<30 ng/mL)	Model I	Model II	Model III	Model IV	Model V
	n (%)	PR (95% CI)	PR (95% CI)	PR (95% CI)	PR (95% CI)	PR (95% CI)
**≤40 years**						
SRQ-20 < 8	75 (70.7)	1.00	1.00	1.00	1.00	1.00
SRQ-20 ≥ 8	84 (79.2)	1.12 (0.96–1.31)	1.12 (0.95–1.31)	1.11 (0.94–1.301)	1.11(0.94–1.31)	1.10 (0.94–1.30)
** *p*-Value ^a^**		0.157	0.163	0.205	0.201	0.226
**>40 years**						
SRQ-20 < 8	40 (70.2)	1.00	1.00	1.00	1.00	1.00
SRQ-20 ≥ 8	31 (88.6)	1.26 (1.02–1.55)	1.27 (1.03–1.55)	1.28 (1.04–1.58)	1.26 (1.01–1.57)	1.25 (1.01–1.56)
** *p*-Value ^a^**		0.028	0.023	0.017	0.037	0.044

^a^ *p*-value for the Wald test for heterogeneity of proportions obtained by means of Poisson regression with robust variance. Model I: Crude analysis (no adjustment). Model II: Analysis adjusted for per capita family income. Model III: Analysis adjusted for Model II + smoking, alcohol consumption, physical activity, and hours of usual sleep. Model IV: Analysis adjusted for Model III + number of pregnancies and work shift. Model V: Analysis adjusted for Model IV + obesity.

## Data Availability

The data presented in this study are available on request from the corresponding author due to privacy.

## Abbreviations

The following abbreviations are used in this manuscript:

CMDs	Common mental disorders
SRQ-20	Self-reporting questionnaire
DSM-5	Diagnostic and Statistical Manual of Mental Disorders, 5th Edition
ICD-10	International classification of diseases, 10th Revision
APMS	Adult psychiatric morbidity survey
ACE	Angiotensin-converting enzyme
PPIs	Proton pump inhibitors
VDBP	Vitamin D binding protein
PR	Prevalence ratio
CI	Confidence interval
KR-20	Kuder–Richardson coefficient
BMI	Body mass index
